# Azelnidipine prevents cardiac dysfunction in streptozotocin-diabetic rats by reducing intracellular calcium accumulation, oxidative stress and apoptosis

**DOI:** 10.1186/1475-2840-10-97

**Published:** 2011-11-04

**Authors:** Vasundhara Kain, Sandeep Kumar, Sandhya L Sitasawad

**Affiliations:** 1 National Centre for Cell Science, NCCS Complex, Pune University Campus, Ganeshkhind Road, Pune-411007, Maharashtra, India

**Keywords:** Azelnidipine, Apoptosis, Ca^2+ ^homeostasis; diabetic cardiomyopathy, mitochondria, oxidative stress

## Abstract

**Background:**

Numerous evidences suggest that diabetic heart is characterized by compromised ventricular contraction and prolonged relaxation attributable to multiple causative factors including calcium accumulation, oxidative stress and apoptosis. Therapeutic interventions to prevent calcium accumulation and oxidative stress could be therefore helpful in improving the cardiac function under diabetic condition.

**Methods:**

This study was designed to examine the effect of long-acting calcium channel blocker (CCB), Azelnidipine (AZL) on contractile dysfunction, intracellular calcium (Ca^2+^) cycling proteins, stress-activated signaling molecules and apoptosis on cardiomyocytes in diabetes. Adult male Wistar rats were made diabetic by a single intraperitoneal (IP) injection of streptozotocin (STZ). Contractile functions were traced from live diabetic rats to isolated individual cardiomyocytes including peak shortening (PS), time-to-PS (TPS), time-to-relengthening (TR_90_), maximal velocity of shortening/relengthening (± dL/dt) and intracellular Ca^2+ ^fluorescence.

**Results:**

Diabetic heart showed significantly depressed PS, ± dL/dt, prolonged TPS, TR_90 _and intracellular Ca^2+ ^clearing and showed an elevated resting intracellular Ca^2+^. AZL itself exhibited little effect on myocyte mechanics but it significantly alleviated STZ-induced myocyte contractile dysfunction. Diabetes increased the levels of superoxide, enhanced expression of the cardiac damage markers like troponin I, p67^phox ^NADPH oxidase subunit, restored the levels of the mitochondrial superoxide dismutase (Mn-SOD), calcium regulatory proteins RyR2 and SERCA2a, and suppressed the levels of the anti-apoptotic Bcl-2 protein. All of these STZ-induced alterations were reconciled by AZL treatment.

**Conclusion:**

Collectively, the data suggest beneficial effect of AZL in diabetic cardiomyopathy via altering intracellular Ca^2+ ^handling proteins and preventing apoptosis by its antioxidant property.

## Background

Individuals with diabetes develop cardiomyopathy independent of coronary artery disease, hypertension or atherosclerosis [1-3]. This 'diabetic cardiomyopathy' is characterized in the early stages by reduced relaxation rates (diastolic dysfunction) while in later stages the systolic dysfunction becomes more prominent [4-6]. Also, hyperglycemia-induced defective intracellular Ca^2+ ^([Ca^2+^]_i_) homeostasis and increased reactive oxygen species (ROS) production have been implicated in this impaired electromechanical performance [7,8]. A combination of these events ultimately leads to diabetic cardiomyopathy [9,10]. Accumulating evidences implicate that ROS plays pivotal role in the pathogenesis of cardiac dysfunction during diabetes, and is likely to be a causative agent for the disturbance in intracellular Ca^2+ ^signaling. Several ion-transport pathways are highly sensitive to redox regulation and oxidative stress directly impedes intracellular Ca^2+ ^homeostasis [11].

In the diabetic heart, abnormal Ca^2+ ^handling during the contractile cycle results in a decreased upstroke phase of the Ca^2+ ^transient due to reduction in the release of Ca^2+ ^from the sarcoplasmic reticulum (SR) by ryanodine receptor (RyR2) [12]. In addition, the diastolic decline of the Ca^2+ ^transient is diminished due to reduced activity of the sarco(endo)plasmic reticulum Ca^2+^-ATPase (SERCA)2a pump [13]. Recent evidences indicate that ventricular dysfunction secondary to myocardial infarction in diabetic rat model was attenuated by restoring the balance of calcium regulatory proteins [14]. As far as the endogenous sources of ROS are considered, NADPH oxidase and mitochondria are the important centers of ROS production and essentially determine the redox state of the myocardium [15-20]. Also, higher myocardial NADPH oxidase activity and increased mitochondrial ROS generation have been detected in diabetes way before diastolic dysfunction is detected indicating a subtle role of hyperglycemia in generation of ROS [21-24]. More importantly, NADPH oxidase activity is markedly increased by high glucose levels [25]. Therefore, improving the abnormal Ca^2+ ^flux in the heart with calcium channel blockers (CCBs) that possesses additional antioxidant property is an attractive strategy to effectively normalize the disturbed Ca^2+ ^transients and improve contractile function.

Long-acting CCBs have been reported to be effective in treating ischemic heart disease; however, their effects on diabetic cardiomyopathy are still unclear. Our previous study showed beneficial effects of AZL in the animal model of STZ-induced diabetes on the circulating markers of cardiac damage, oxidative stress, homocysteine, pro- and anti-inflammatory cytokines [26]. The present study was designed to examine the effect of AZL on cardiomyocyte contractility and intracellular Ca^2+ ^homeostatic defects in the streptozotocin (STZ)-diabetic rat model with special relevance to oxidative stress and apoptosis.

## Methods

### Development and characterization of diabetic rats

Six to eight-week-old male Wistar rats (NCCS, Pune, India), weighing 250 to 280 g, were made diabetic by single intra-peritoneal (IP) injection of streptozotocin (STZ) (55 mg/kg, Sigma, St. Louis, MO). Control animals were treated with vehicle (0.1 mol/L sodium citrate buffer, pH 4.5). Hyperglycemia (blood glucose > 200 mg/dL) was confirmed 3 days post STZ injection using a glucometer (AccuCheck; Roche, Germany). Diabetic animals were treated with single dose of 5 mg/kg AZL suspended in 1% carboxy methyl cellulose, administered orally by gavage, starting the 4^th ^day of STZ treatment (n = 12) daily for a period of 12 weeks. Blood glucose and body weight were measured weekly and at the end of the study. All procedures were approved by Institutional Animal Care and Use Committee and were performed in accordance to the standards for the care and use of animal subjects, as stated in the Guide for the Care and Use of Laboratory Animals (Institute of Laboratory Resources, National Academy of Sciences, Bethesda, MD).

### Measurement of cardiac contractility *in vivo*

Urethane (1 g/kg bw IP) was selected as an anesthetic agent as its single dose induces long-term anesthesia and analgesia with minimal cardiovascular and respiratory system depression [27]. The right carotid artery was cannulated with a microtip pressure transducer (SPR-671, Millar Instruments) connected to 8-channel PowerLab instrument via bridge amplifier (AD Instrument). The pressure-tip transducer catheter was then advanced into the left ventricle for the evaluation of ventricular pressures. LV systolic and end-diastolic pressures, the maximum rate of LV systolic pressure rise (+ΔP/Δ*t*_max_) and minimum rate of LV systolic pressure decay (-ΔP/Δ*t*_min_) were monitored and recorded using Chart 5.5. Rectal temperature was maintained at 36-38°C throughout the procedure [28].

### Measurement of cell shortening/relengthening and intracellular Ca^2+ ^fluorescence in isolated cardiac myocytes

Adult rat ventricular myocytes (ARVMs) were isolated as described previously [29]. The mechanical properties of ventricular myocytes from control and treated rats were assessed using a SoftEdge MyoCam system (IonOptix Corp., Milton, MA, USA) [29]. Cell shortening and relengthening were assessed using the following indices: peak shortening (PS)--indicative of peak ventricular contractility, time to PS (TPS)--indicative of contraction duration, time to 90% relengthening (TR_90_)--representing cardiomyocyte relaxation duration, and maximal velocities of shortening (+dL/dt) and relengthening (-dL/dt)--indicators of maximal velocities of ventricular pressure rise/fall. Briefly, myocytes were loaded with Fura-2AM (0.5 μM) for 10 min and fluorescence measurements were recorded. Resting calcium, qualitative changes in the intracellular calcium, and fluorescence decay time (Tau) were also measured. Both single- and bi-exponential curve-fit programs were applied to calculate the intracellular Ca^2+ ^decay constant [29]. At least 25 individual myocytes were used for data collection. Changes in [Ca]_i _were calculated by determining the rise in [Ca]_i _relative to basal levels measured immediately before that particular experimental maneuver.

### Determination of intracellular superoxide (O_2_·^-^) levels in diabetic ARVMs

ROS generation was measured using fluorescent probe DHE, an O_2_·^-^-sensitive probe [30,31]. DHE at a final concentration of 2 μM was added to the ARVMs from control and diabetic rat, and the staining was carried out at 37°C. The cells were washed using phosphate-buffered saline (PBS) and fixed with 4% buffered paraformaldehyde. The coverslip was mounted with antifade on a glass slide and observed using a confocal laser-scanning microscope (Zeiss 510; Zeiss GmbH, Oberkochen, Germany). Quantitative determination of DHE fluorescence was done using fluorimetry. Briefly, post treatment, the cells were washed with PBS, and re-suspended in HEPES buffer (5 mM HEPES, pH 7.4; 5 mM KCl, 140 mM NaCl, 2 mM CaCl_2_, 1 mM MgCl_2 _and 10 mM glucose), stained with DHE for 20 min and their fluorescence intensities were acquired by fluorimetery (SpectraMaxPro, USA).

### Western blot analysis

Ventricular tissue was homogenized into radioimmunoprecipitation assay lysis buffer (120 mM NaCl, 1.0% Triton X-100, 20 mM Tris-HCl, pH 7.5, 10% glycerol, 2 mM EDTA, protease inhibitor cocktail (Roche GmbH, Germany) and the protein concentration for each sample was determined using a Bradford-based protein assay kit (Bio-Rad, Hercules, USA). For immunoblotting, 50-60 μg of protein lysate per sample was denatured in 2× SDS-PAGE sample buffer and resolved on SDS-PAGE (4% to 10%), transferred to a PVDF membrane (Millipore, Germany), blocked with non-fat milk, and probed for Troponin I (Cell Signaling, USA), RYR2, Mn-SOD (Sigma), Bcl-2 and p-67^phox ^(Santa Cruz Biotechnology, Inc, USA), SERCA2 ATPase (Affinity Bioreagents, USA) and β-actin (ICN Biomedicals Inc. USA) and HRP-conjugated appropriate secondary antibody (Bio-Rad, Hercules, USA). The enhanced chemiluminescence was detected using chemiluminescence detection system (Pierce Chemical, Rockford, IL, USA). Membranes were stripped and reprobed with β-actin (ICN Biomedicals, USA) primary antibody (1:10, 000) as a protein loading control.

### Terminal Transferase dUTP Nick End Labeling (TUNEL) Assay

Apoptotic cell death in cardiomyocytes in heart was detected by *in situ *terminal deoxynucleotidyl transferase-mediated dUTP nick-end labeling. TUNEL staining was performed on the cardiac tissue sections using the fluorescent In situ Cell Death Detection Kit (Roche Diagnostic GmbH, Mannheim, Germany) according to the manufacturer's instructions. TUNEL-positive nuclei were counted in a minimum of 150 cells per group by fluorescence microscopy and an apoptotic index (AI) was determined as the percentage of TUNEL-positive nuclei which was scored blindly by two evaluators. The statistical analysis revealed a good correlation (Pearson's correlation coefficient 0.91, p < 0.0001).

### Statistical analyses

At least six to seven rats were used per group for each treatment (control and diabetic with or without AZL) for mechanical and intracellular Ca^2+ ^recordings. For each experimental series, data are presented as means ± SE. Statistical significance (*P <*0.05) for each variable was estimated by ANOVA by using Tukey-Kramer post tests using Prism 4.0 GraphPad software (GraphPad, *San Diego*, CA, USA). A *p *value less than 0.05 was considered statistically significant.

## Results

### AZL improves cardiac contractile dysfunction in diabetic rat heart

#### I) *In vivo*

STZ-induced diabetic animals showed stable signs of diabetes, including hyperglycemia, reduced levels of insulin. Also there was a noted increase heart/body weight ratio (H/BW). Diabetic rats treated with AZL showed improvement in these physiological parameters. STZ group showed reduced rate of contraction (+ Δp/Δt) and rate of relaxation (- Δp/Δt) as compared with control group. A significant reduction in the heart rate and impairment in left ventricular pressure (LVP) as well as left ventricular end diastolic pressure (LVEDP) was observed in STZ-diabetic animals. Table 1 shows AZL-treated diabetic rats significantly improved left ventricular parameters.

**Table 1:** Hemodynamic parameters from control, diabetic rats and diabetic rats treated with AZL (5 mg kg^-1 ^day^-1^)

Parameters	Control (n = 10)	STZ* (n = 10)	AZL** (5 mg/kg/day) (n = 12)
Systolic Pressure (mm Hg)	138.1 ± 4.7	93.8 ± 4.1	124.6 ± 8.9

Diastolic Pressure (mm Hg)	85.8 ± 6.2	64.0 ± 3.5	82.5 ± 7.1

Systolic duration (s)	0.085 ± 0.01	0.140 ± 0.01	0.084 ± 0.01

Diastolic duration (s)	0.117 ± 0.02	0.297 ± 0.02	0.127 ± 0.01

Heart rate (BPM)	323.3 ± 20.9	244.9 ± 10.8	312.2 ± 14.1

LV+dp/dt (mm Hg/s)	5861.4 ± 729.5	3219.3 ± 297.8	6098.6 ± 395.4

LV-dp/dt (mm Hg/s)	-6846.8 ± 752.1	-3553.0 ± 437.3	-7015.9 ± 391.5

#### II) In isolated single myocytes from diabetic rat

The contractile dysfunction observed *in vivo *could be partly due to extrinsic factors, such as changes in circulating metabolites or hormones. In isolated myocytes, the influence of extrinsic factors is eliminated, which allows for the evaluation of intrinsic contractile dysfunction. Therefore, contractile function was examined in isolated myocytes from control, diabetic and AZL-treated groups.

**Figure 1:**
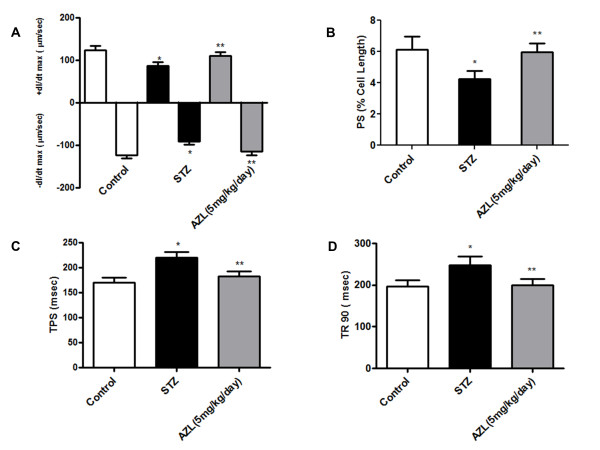
**Contractile properties of cardiomyocytes isolated from control, diabetic rats and diabetic rats treated with AZL (5 mg kg^-1 ^day^-1^)**. *A*: Maximal velocity of shortening (+dL/dt, *A*) and relengthening (-dL/dt, *B*) of ventricular myocytes isolated from control, diabetic rats and diabetic rats treated with AZL (5 mg kg^-1 ^day^-1^). B. Graph illustrates Peak shortening (PS, normalized to cell length) of the myocytes isolated from control, diabetic rats and diabetic rats treated with AZL. C. Graph illustrates time-to-peak shortening (TPS) of the myocytes isolated from control, diabetic rats and diabetic rats treated with AZL. D. Graph illustrates time-to-90% relengthening (TR_90_) of the myocytes isolated from control, diabetic rats and diabetic rats treated with AZL. Values are means ± SE; *n *= 151-163 cells from 5-7 rats per group, *P < 0.05 vs. control group; **P < 0.05 vs. diabetic group.

Peak shortening (PS) amplitude normalized to cell length was significantly decreased in ventricular myocytes under STZ-induced diabetes (27.82% ± 8.17, p < 0.05). Myocytes from the diabetic group also demonstrated significantly prolonged time-to-peak shortening (TPS, (29.30% ± 11.2, p < 0.05) and time-to-90% relengthening (TR_90_, (25.72% ± 20.67, p < 0.05) compared with control. AZL treatment completely abolished the diabetes-induced abnormalities of PS, TPS and TR_90 _(Figure 1A-D). The maximal velocities of shortening (+dl/d*t*) and relengthening (-dl/d*t*) were significantly reduced by diabetes and AZL treatment restored the diabetes-induced dysfunction (Figure 1A and 1B). Myocytes isolated from 12-week AZL-treated diabetic rats had significantly smaller deviation from corresponding values when compared to the myocytes isolated from the control group suggesting role of AZL in maintaining ventricular function of the heart along with preserving the contractile properties of individual myocytes.

### AZL maintains global Ca^2+ ^homeostasis in diabetic rat heart

**Figure 2:**
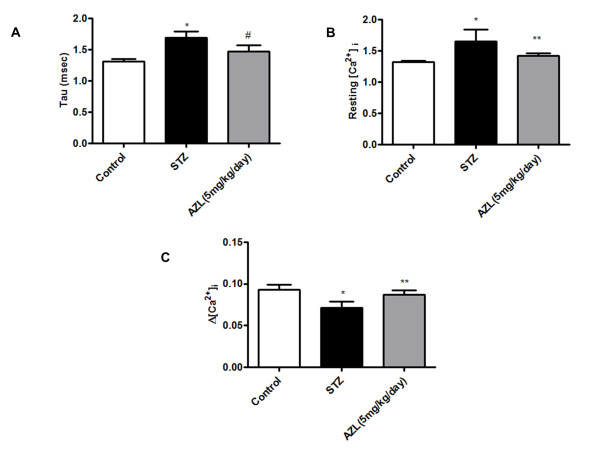
**Intracellular Ca^2+ ^transient properties of ventricular myocytes isolated from control, diabetic rats and diabetic rats treated with AZL (5 mg kg^-1 ^day^-1^)**. *A*: Baseline intracellular Ca^2+ ^concentrations; *B*: Increase in intracellular Ca^2+ ^transient in response to electrical stimulus; *C*: rate of cytosolic free Ca^2+ ^decrease (Tau). Values are means ± SEM; *n *= 104 to 126/group. *P < 0.05 vs. control group; **P < 0.05 vs. diabetic group.

Our data indicated enhanced level of resting intracellular Ca^2+ ^in STZ induced diabetic rat myocytes. The rise of intracellular Ca^2+ ^in response to electrical stimuli was significantly reduced. "Diabetic" myocytes showed reduced intracellular Ca^2+ ^clearing rate (single and bi-exponential decay). Furthermore, 12-weeks of AZL treatment significantly ablated intracellular Ca^2+ ^abnormalities in STZ treated diabetic rats. Consistent with its response in cardiomyocyte shortening, AZL treatment improved diabetes induced changes in Ca^2+ ^homeostasis including elevated resting intracellular Ca^2+ ^levels, depressed intracellular Ca^2+ ^rise in response to electrical stimuli and prolonged intracellular Ca^2+ ^decay (Figure 2 A-C).

### AZL reduces superoxide (O_2_·^-^) from diabetic ARVMs

**Figure 3:**
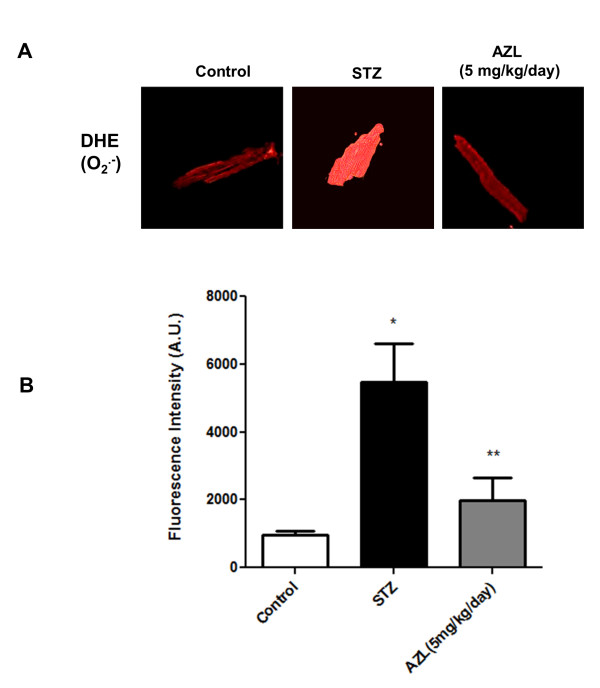
**Generation of O_2_·^- ^in ventricular myocytes isolated from control, diabetic rats and diabetic rats treated with AZL (5 mg kg^-1 ^day^-1^)**. A. Representative confocal laser scanning microscopy images of cells fluorescently stained with DHE. B. Graph shows quantification of DHE fluorescence emission (Arbitrary Units) of the myocytes isolated from control, diabetic rats and diabetic rats treated with AZL after staining with DHE. Data represents Means ± SEM; *n *= 104 to 126/group. *P < 0.05 vs. control group; **P < 0.05 vs. diabetic group.

Superoxide overproduction in the cellular systems is an important feature of diabetic cardiomyopathy. A significant increase in the DHE fluorescence was observed in isolated myocytes from the STZ diabetic rats indicating generation of superoxide radicals in comparison to the myocytes isolated from control rats. Myocytes from 12-week AZL-treated diabetic rats showed significant decrease in the fluorescent levels indicating that AZL can reduce the superoxide production (Figure 3A).

Qualitative analysis of DHE fluorescent intensity showed that there was a 5.6 ± 0.5 fold (p < 0.001) increase in the DHE fluorescence in the "diabetic" myocytes when compared to the "control" myocytes. The fluorescence intensity of DHE showed a 2.7 ± 0.8 fold (p < 0.001) decrease in the AZL treated group when compared to the STZ diabetic rats (Figure 3B). These results suggest that AZL treatment prevents diabetes-induced accumulation of superoxide in the myocytes.

### AZL neutralizes the increased expression levels of contractile proteins (Troponin I) in diabetic heart

**Figure 4:**
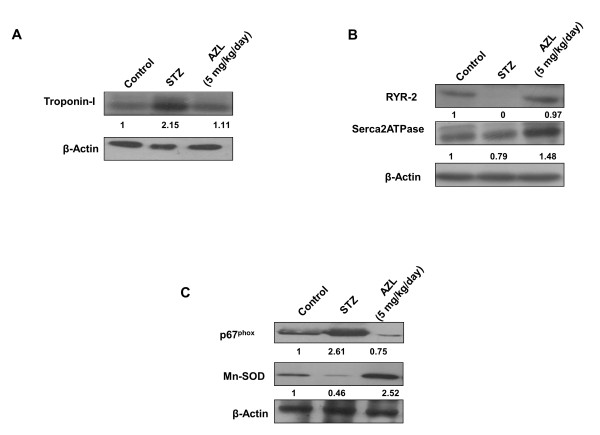
**Western blot showing altered expression of calcium and ROS regulatory proteins in control, diabetic rats and diabetic rats treated with AZL (5 mg kg^-1 ^day^-1^)**. *A: *Troponin I, B: RyR-2 and SERCA2ATPase, C: p67^phox ^and Mn-SOD. Image is representative of the best of three separate experiments. β-actin served as loading control. Normalized densitometric quantification has been depicted by numerical value below the bands.

Since mechanical dysfunction that characterizes diabetic cardiomyopathy plays an essential role in the Ca^2+ ^regulation of muscle contraction, we studied the effect of AZL on the expression level of cardiac Troponin I in our experimental model. Our western blot results showed a 2.15-fold increase (p < 0.05) in the expression of Troponin I in the heart of STZ treated animal when compared with the control. The increased cardiac Troponin I expression counter-balanced in the cardiac tissue from AZL treated diabetic rats (Figure 4A). These findings suggest that AZL treatment under diabetic condition prevents cardiac damage by reducing the expression of cardiac Troponin I.

### AZL regulates the expression of calcium regulatory receptors and channels in diabetic heart

Downregulation of key Ca^2+^-handling proteins like sarco(endo)plasmic reticulum Ca^2+^-ATPase (SERCA)2a and ryanodine receptor (RyR2) is one of the major cause of abnormal Ca^2+ ^homeostasis in diabetic cardiomyopathy [13,32-34]. The alteration in SERCA2a and RyR2 expression results in altered cytosolic Ca^2+ ^transients, leading to abnormal contraction. Our western blot results indicated a complete loss of expression of RYR2 and a significant reduction in SERCA-2a in STZ-treated diabetic rats compared to the controls (Figure 4B). Reduction in the RyR2 expression induced by uncontrolled diabetes was attenuated with AZL treatment. Similarly, the expression of SERCA- 2a was also restored after AZL treatment (Figure 4B). These findings indicate that AZL treatment under diabetic condition prevents dysregulation of calcium regulatory proteins in the heart.

### AZL induces downregulation of NADPH oxidases and prevents oxidative stress

We determined effect of AZL treatment on the endogenous pro-oxidants and antioxidants like p67^phox ^and Mn-SOD. Our western blot results showed increased expression of p67^phox ^in diabetic heart. This indicated that endogenous pro-oxidant system was triggered under diabetic condition which may further aggravate oxidative stress. Treatment with AZL significantly attenuated the p67^phox ^expression (Figure 4C). On the other hand, a significant decrease (0.46 fold, p < 0.05) in the expression of Mn-SOD in STZ-diabetic heart was observed as compared to control hearts. Treatment with AZL resulted in a 2.5-fold (p < 0.05) increase in the expression of Mn-SOD in the diabetic condition. These results indicate that AZL exerts its protective effects by targeting the NADPH oxidase and mitochondrial redox enzymes (Figure 4C).

### AZL prevents STZ-induced cardiac apoptosis

**Figure 5:**
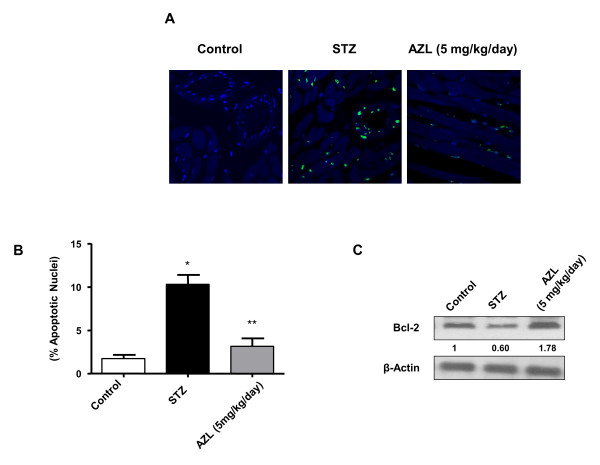
**Apoptosis in diabetic rat hearts and treated with AZL**. A: representative photographs of *in situ *detection of apoptosis in heart tissue from controls, diabetic rats treated with vehicle, and diabetic rats treated with AZL (5 mg kg^-1^day^-1^). Total nuclei were labeled with DAPI (blue), and apoptotic nuclei were detected by TUNEL staining (green). B: average number of percent TUNEL-positive nuclei in tissue sections from each group (*n *= 4 to 5 sections each per group). *P < 0.05 vs. control group; **P < 0.05 vs. STZ treated group. C: Western blot showing bcl-2 expression in hearts from the same groups. Image is representative of the best of three separate experiments. β-actin served as loading control. Normalized densitometric quantification has been depicted by numerical value below the bands.

Apoptosis was also evaluated in the cardiac tissue by TUNEL assay. Diabetic rats showed significant myocardial apoptotic cell death manifested by a 6 fold increase in the percent TUNEL-positive cell labeling compared with control rats (Figure 5A and 5B). The counts of TUNEL-positive nuclei significantly decreased in AZL-treated group. Under oxidative stress, mitochondria play an important role in apoptosis and a decrease in the level of bcl-2 is observed. This decreasing bcl-2 expression is one of the hallmarks of apoptosis through mitochondrial pathway. STZ diabetic animal showed elevated cardiac apoptosis, as indicated by decreased bcl-2 protein expression, compared to the control animals. AZL-treated diabetic rats expressed enhanced level of bcl-2 in the heart lysates indicating that AZL plays protective role in cardiac apoptosis.

## Discussion

The key findings of our present study demonstrated that AZL treatment for 12 weeks in diabetic animal inhibits the development of early characteristics of diabetic cardiomyopathy, such as, prolonged relaxation and abnormal E-C coupling *in vivo *in the intact myocardium as well as in the isolated ventricular myocytes. Delayed diastolic relaxation in diabetic cardiomyopathy is related to diminished removal of [Ca^2+^]_i _from cardiomyocytes after the systolic contraction event. Treatment with AZL showed improvement in the systolic and diastolic duration. Also the markers for diastolic dysfunction, viz., maximal rise and decay in the blood pressure showed improvement. These mechanical abnormalities may be underscored by altered intracellular Ca^2+ ^homeostasis that was associated with enhanced oxidative stress. We found that AZL prevents maladaptive ventricular remodeling. Untreated diabetes further accelerated oxidative stress at molecular level by upregulating the endogenous NADPH oxidases like p67^phox ^and downregulation of Mn-SOD. Treatment with AZL normalized the p67^phox ^and Mn-SOD expression. Also, AZL treatment normalized the levels of cardiac troponin I, RyR2, and SERCA2a. The ability of AZL to restore all the parameters to the control level provides a plausible explanation for its ability to prevent diabetes-induced defects in calcium signaling. The intrinsic antioxidant activity of AZL might thus contribute to its beneficial effects on LV dysfunction and cardiac failure.

Abnormalities in the myocardial calcium handling can contribute to deranged cardiac mechanics in the diabetic heart. Diabetes impairs sarcoplasmic reticular calcium pump activities, which reduces the rate of Ca^2+ ^removal from the cytoplasm in diastole [7]. Such alterations may contribute to the increased diastolic stiffness characteristic of diabetic cardiomyopathy. Intracellular retention of calcium in diabetes is associated with the depletion of high-energy phosphate stores, derangement of cellular ultrastructure and can lead to cardiac dysfunction. Calcium channel blockers in conjunction with antioxidants can reverse the intracellular calcium defects and prevent diabetes induced myocardial changes. The present study investigates Ca^2+^-dependent regulation of cellular function in diabetic cardiomyocytes and highlights role of AZL in prevention of early onset of diastolic dysfunction at cellular and organ level.

AZL is a novel dihydropyridine calcium channel blocker which has a long lasting anti-hypertensive action [35]. It is generally well tolerated and its use is not associated with reflex tachycardia [35]. AZL has recently been approved in Japan for the treatment of patients with hypertension. Very recently, the results from OSCAR trial revealed that regimen that included AZL showed less composite fatal and nonfatal cardiovascular events compared to the group treated with other CCBs like amlodipine. Another study demonstrated the novel beneficial aspect of azelnidipine, whereby azelnidipine could play a protective role against atherosclerosis by suppressing monocyte chemoattractant protein-1 overexpression in endothelial cells [36]. Very recently, azelnidipine treatment have been shown to be useful in conditions like glucose tolerance, insulin sensitivity, inflammation, and number of circulating progenitor cells in non-diabetic patients with essential hypertension [37]. Another comparative study between azelnidipine and olmesartan revealed that AZL was equally effective in reducing the blood pressure but also reduced the urinary albumin/creatinine ratio and 8-hydroxydeoxyguanosine and renal fatty acid binding protein levels significantly compared with the amlodipine group. Also, AZL group showed lower plasma aldosterone levels indicating that AZL is far more effective in preventing albuminuria and oxidant stress in hypertensive diabetic patients with CKD than amlodipine [38]. In the settings of oxidative stress-induced hepatotoxicity mice model, Azelnidipine significantly decreased inflammatory cell infiltration, profibrotic gene expressions, Hematopoietic stem cell activation, lipid peroxidation, oxidative DNA damage and fibrosis and also prevented the decrease in the expression of antioxidant enzymes [39]. It has been now well known that AZL can produce some beneficial effects independent of its anti-hypertensive effect [26], so the direct pharmacological effect of AZL on the initial management and prevention of diabetic cardiomyopathy are paid more attention.

Our study was an attempt to identify the effect of AZL on alteration of cardiomyocyte contraction and related calcium regulatory proteins, which might explain the effect of AZL on cardiac performance under diabetic conditions.

Diabetes is characterized by consistently elevated blood glucose levels, decreased insulin levels and increased heart/body weight ratio indicative of hypertrophied heart was observed in the STZ diabetic rat. Cardiac hypertrophy involves remodeling of entire heart especially in the left ventricular region which eventually leads to impaired diastolic function, further causing deterioration of cardiac morphology and function. The important finding of the present study is that STZ-induced hyperglycemia leads to dilapidated cardiac function further leading to diabetic cardiomyopathy. Our STZ diabetic rats showed left ventricular dysfunction. This study along with our previous report [26] provides evidence that hyperglycemia-induced left ventricular dysfunction due to oxidative stress induced by reactive oxygen species (ROS) and reactive nitrogen species (RNS) and defective antioxidant system contributing to the development of cardiomyopathy. Apoptosis induced by hyperglycemia is an early event in the pathophysiology of diabetic cardiomyopathy [40]. Hyperglycemia and insulin resistance independently contribute to functional alteration in the heart [41-44]. AZL treatment in streptozotocin diabetic rats has been shown to improve these functional cardiac abnormalities perhaps through tyrosine kinase-dependent increases in intracellular [Ca^2+^]_i _removal after systole. In the present study, treatment with AZL showed improvement in the systolic and diastolic duration. Also the markers for diastolic dysfunction, viz., maximal rise and decay in the blood pressure showed improvement. In the present study, we found that AZL prevents ventricular remodeling accompanied by cardiac dysfunction. We also demonstrated that AZL did not alter blood pressure and this suggests that AZL has preventive effects on cardiac dysfunction beyond its antihypertensive effects. Oxidative stress might play an important role in the progression of LV dysfunction and failure, the data somewhat is consistent with previous finding using STZ diabetic models [45-47]. These mechanical abnormalities may be underscored by altered intracellular Ca^2+ ^homeostasis that was associated with enhanced oxidative stress. The intrinsic antioxidant activity of AZL might thus be a contributor to its beneficial effects on LV dysfunction in diabetic cardiomyopathy. Although these findings are of interest, no clinical trials to date have investigated the effect of AZL on the development and progression of congestive heart failure in diabetic patients.

In our study, certain diabetes-induced mechanical defects were not improved or protected by AZL treatment. For example, AZL improved the diabetes-induced reduction in PS but not Ca^2+^-induced Ca^2+ ^release. Although the underlying mechanism is largely unknown, the ability of AZL to enhance myofilament Ca^2+ ^sensitivity may play a role. This is somewhat supported by the results shown in Figure 1, where myocytes from the AZL-treated group exhibit an improved PS compared with the myocytes from the diabetic group. The results from this study revealed that AZL treatment lowered the resting intracellular Ca^2+ ^levels in the diabetic group. This AZL-induced reduction in resting intracellular Ca^2+ ^level may be associated with an enhanced SERCA Ca^2+ ^clearing ability in the AZL-treated group (Figure 2) and is consistent with the vasodilatory and cardioprotective effect against Ca^2+ ^overload under pathological conditions such as heart failure. The frequency-PS relationship was improved by AZL-treated diabetic group (Figure 1), indicating a preserved sarcoplasmic reticulum (SR)-replenishing function in diabetic hearts. One possible explanation is that AZL may significantly augment the basal SR Ca^2+ ^load in the diabetic group. The impaired intracellular Ca^2+ ^homeostasis may be associated with a reduction in the main Ca^2+^-regulating protein SERCA2 and ryanodine receptor (RyR) proteins indirectly with reduced levels of Troponin I under the diabetic state [48]. Interestingly, the STZ diabetes-induced oxidative stress, apoptosis and alterations in oxidative stress-related signaling molecule p67^phox ^NADPH oxidase were effectively alleviated by AZL treatment. It also improved the levels of Troponin I, RyR2, and SERCA2a. Because SERCA and RYR2 contributes to ~92% of the cytosolic Ca^2+ ^removal workload in rat hearts [49], our finding of an overt reduction in SERCA2a protein level in STZ-induced diabetic hearts should have provided one of the most compelling explanations for the slowed intracellular Ca^2+ ^clearing and prolonged duration of relaxation (TR90). The ability of AZL to restore all the parameters to the control level provides a plausible explanation for its ability to prevent diabetes-induced defects in calcium signaling. Further, restoration of TPS after AZL treatment indicates that AZL may have a significant effect on the key rate-limiting components determining the length of contraction duration such as SR Ca^2+ ^release, troponin, tropomyosin, and actin-myosin cross-bridge linking. These observations are consistent with the functional data of improved intracellular Ca^2+ ^clearing and duration of relengthening (TR_90_) after AZL treatment. These results suggest that AZL treatment may ameliorate contractile disturbances in cardiomyocytes from diabetic animals and could provide therapeutic potential in the treatment of diabetic cardiomyopathy.

Since AZL did not affect the hyperglycemic condition in diabetes, our data suggest that STZ-induced diabetes may elicit cardiac contractile dysfunction and intracellular Ca^2+ ^mishandling likely through enhanced oxidative stress and cell injury.

Increased oxidative stress is believed to be an initial and important step in the development of cardiac dysfunction and cardiomyopathy. NADPH oxidase and mitochondria are considered to be important sources of ROS [15-20] and are critical determinants of the redox state of the diabetic myocardium. Previous studies reported that membrane translocation of p67^phox ^and the increased expression of p22^phox ^was prevented by N-acetyl L cysteine [50]. Therefore, we further tested whether AZL exerts its antioxidative properties by modulating the expression and function of NADPH oxidase subunit p67^phox ^and mitochondrial ROS-eliminating enzyme Mn-SOD. The results from the isolated cardiomyocytes study showed that hyperglycemia leads to increased oxidative stress by enhancing the O_2_·^- ^generation, by decreasing the expression of antioxidant enzyme Mn-SOD and by increasing expression of p67^phox^. The main new findings of this study are that AZL treatment prevents the increased expression of p67^phox^, and enhances Mn-SOD expression, thus reducing myocardial superoxide formation in the diabetic rat hearts. This reduction of O_2_·^- ^generation and normalization of p67^phox ^and Mn-SOD after AZL treatment indicate that AZL reduces diabetic cardiac damage by targeting the redox signaling pathways. Moreover, an increase in the expression of p67^phox ^and decrease in the expression of Mn-SOD as well as bcl-2 and normalization of these expressions by AZL indicates a mutual functional relationship between NADPH oxidase and mitochondria. AZL in our study not only improves cardiac contractile function but also offers protection against oxidative stress, apoptosis and ultimately leading diabetic cardiomyopathy.

### Limitations of the study

We did not test the effect of known antioxidants in comparison to AZL in the present study. We observed the changes after the treatment duration of 12 weeks. Some of the changes could be result of the overall functional improvement due to AZL treatment and may not be directly attributable to AZL treatment. The hemodynamic parameters were evaluated at the end of the study, and comparisons were made with comparing with diabetic and non-diabetic control in the experimental design. As a result of which the signals were recorded only at the end of the experimental period, leading to the lack of baseline values in the same animals at the start of the study.

## Conclusion

In conclusion, the present study reveals the beneficial effects of AZL treatment on diabetes induced early left ventricular dysfunction. AZL exhibited additional antioxidant properties in addition to its calcium channel blocking activity. This intrinsic antioxidant property of AZL may provide a promising advantage over other calcium channel blockers in the management of compromised heart function especially under diabetes.

## Competing interests

The authors declare that they have no competing interests.

## Authors' contributions

VK and SK contributed equally to the experimental designing and bench work. SLS conceived and designed the study. All authors have read, discussed and approved the final manuscript.
